# Similarity of vocational interest profiles within families: A person‐centered approach for examining associations between circumplex profiles

**DOI:** 10.1111/jopy.12418

**Published:** 2018-08-30

**Authors:** Julian M. Etzel, Oliver Lüdtke, Jenny Wagner, Gabriel Nagy

**Affiliations:** ^1^ Leibniz Institute for Science and Mathematics Education Kiel Germany; ^2^ Centre for International Student Assessment Kiel, Munich, and Frankfurt Germany; ^3^ University of Hamburg Hamburg Germany

**Keywords:** circumplex model, couple similarity, interest profile similarity, parent–child similarity, structural summary method

## Abstract

**Objective:**

Our study addressed three questions concerning the similarity of vocational interests within families: (a) How similar are vocational interests of mothers and fathers? (b) How similar are vocational interests of parents and their children? (c) Is the inference about parent–child profile similarity affected by mother–father profile similarity?

**Method:**

Data from *N*=1,624 tenth graders and their parents were used to analyze interest profile similarity by means of a pseudo‐coupling approach. Similarity was assessed on the level of observed profiles and model‐based circumplex profiles.

**Results:**

Interest profiles of mother–father and parent–child dyads were more similar to each other than those of corresponding arbitrarily paired dyads. However, when the similarity between the parents’ interest profiles was accounted for, only same‐sex parent–child dyads were more similar to each other than would be expected by chance. All findings were mirrored on the level of observed profiles and model‐based circumplex profiles.

**Conclusions:**

In sum, our findings support the validity of the circumplex model of vocational interests and emphasize the benefits of explicitly considering its implications when analyzing profile similarity. Moreover, we were able to show that the statistical evaluation of profile similarities must account for normative profile components.

## 1 INTRODUCTION

For decades, vocational interests have been a central topic in organizational, educational, and personality research. While the majority of research is concerned with the structure and stability of vocational interests (e.g., Low, Yoon, Roberts, & Rounds, 2005; Tracey & Rounds, 1993), with their predictive power for academic success, choice behavior, and life events (e.g., Nye, Su, Rounds, & Drasgow, 2017; Stoll et al., 2016; Van Iddekinge, Roth, Putka, & Lanivich, 2011), as well as with associations between interests, personality traits, and abilities (e.g., Ackerman & Heggestad, 1997), little is known about intrafamily associations of vocational interests.

This comes as a surprise because there are at least two kinds of relationships within families that could be of particular interest to different fields of psychological research. First, associations between parents’ vocational interests might foster our understanding of couple similarity. Although it has been shown that individuals choose romantic partners who have similar psychological characteristics (e.g., Luo, 2017), little to nothing is known about the role that vocational interests play in partner selection. Second, associations between interests of parents and those of their children might deepen our understanding of vocational interest development. Although most theories emphasize the importance of social learning, role orientation, and genetic contributions (Holland, 1997; Lent, Brown, & Hackett, 1994), empirical findings concerning actual parent–child interest similarity are scarce and equivocal.

In recent years, vocational interest research methodology has advanced considerably. For example, researchers have begun to explicitly consider the structural implications of Holland's (1997) model of vocational interests: the circumplex (e.g., Nagy, Trautwein, & Maaz, 2012; Tracey, Wille, Durr, & De Fruyt, 2014). Using the so‐called structural summary method (SSM; Gurtman & Balakrishnan, 1998), it is possible to reduce the multivariate interest profile to three theoretically meaningful components: *profile elevation*, the mean of the individual's profile scores; *profile differentiation*, an indicator of intra‐profile variability; and *profile orientation*, a measure of profile direction, indicating the kinds of activities a person is primarily interested in. Furthermore, personality researchers have recently underscored the need to consider normative profile components, especially in the analysis of profile similarity (e.g., Borkenau & Leising, 2016; Furr, 2008).

While researchers concerned with the aforementioned phenomena (e.g., interest congruence and choice behavior) have already begun debating and updating their findings in light of these methodological advances, these advances have hardly ever been considered in research on intrafamily interest similarity. In our study, we addressed this research gap by showing that these methodological advances have important consequences for the interpretation and statistical evaluation of interest profile similarity. Specifically, we employed a person‐centered pseudo‐couple approach to analyze (a) the extent to which vocational interests of mothers and fathers are similar to each other, (b) the extent to which vocational interests of parents and those of their children are similar to each other, and (c) whether or not the inference about parent–child interest similarity is affected by mother–father interest similarity. All research questions were examined on both the level of observed interest profiles as well as on the level of model‐based circumplex profiles.

In the following sections, we will first introduce the circumplex model of vocational interests and its implications for individual interest profiles. We will then demonstrate that this structural model also poses as a model for assessing the similarity between interest profiles via profile correlations, before turning our attention to methodological challenges of analyzing such similarities. Finally, we will briefly review possible explanations and existing empirical evidence for interest similarity within families in light of these methodological arguments.

### 1.1 Holland's model of vocational interests

Holland (1997) considers vocational interests to be an integral part of the human personality and defines them as relatively stable psychological representations of activity preferences. He postulates six distinct interest types: Realistic, Investigative, Artistic, Social, Enterprising, and Conventional (RIASEC). The Realistic type is characterized by preferences for manual tasks and working with machines. Individuals categorized as Investigative prefer researching, favor exploratory and analytic activities, and have an aversion to repetitive tasks. Artistic individuals prefer creative, unsystematic, and ambiguous activities. The Social type is characterized by a preference for informing, helping, or training other people. The Enterprising type is characterized by a preference for manipulating and leading others. Finally, people categorized as Conventional prefer activities that involve the systematic and structured manipulation of data (Holland, 1997).

According to Holland, the vocational personality pattern or profile (i.e., the set of scores on the six RIASEC dimensions) and its fit with the environment are the central determinant of vocational choice behavior and success (Holland, 1997; Volodina, Nagy, & Retelsdorf, 2015). Therefore, vocational interests are inherently suited for the application of person‐centered or idiographic approaches (PCAs). PCAs stand in contrast to the more conventional variable‐centered or nomothetic approaches (VCAs; Block, 1961), which are primarily used to examine associations between variables in the population (e.g., the correlation between fathers’ and daughters’ Realistic scale scores). As such, VCAs yield information about the associations between the rank order of two interest trait scores over persons, and not about the actual similarity of interest profiles between two persons. On the other hand, PCAs consider entire multivariate trait configurations, thus making it possible to assess similarity between pairs of individuals instead of pairs of variables.

**Figure 1:**
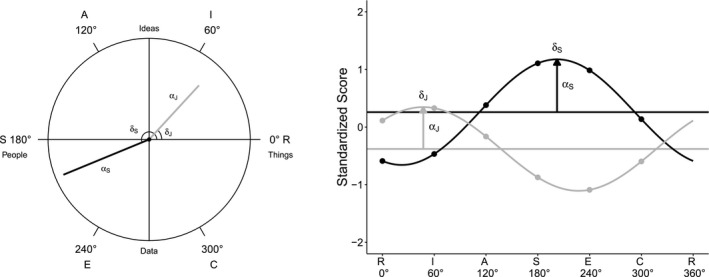
Circumplex projection and model‐based circumplex profiles of an exemplary mother–son dyad. *Notes*. R=Realistic; I=Investigative; A=Artistic; S=Social; E=Enterprising; C=Conventional; *α*=profile differentiation; *δ*=profile orientation of mother, Sandy (S, black), and son, Jeremy (J, gray)

Another central aspect of Holland's (1997) theory is the assumption that the six interest types display a specific structure: They are distributed around the perimeter of a circumplex (left side of Figure 1). Thereby, the proximity between two interest types mirrors their psychological similarity. This so‐called calculus hypothesis has been supported in various groups, contexts, and cultures (Nagy, Trautwein, & Lüdtke, 2010; Tracey & Rounds, 1993). In the subsequent sections, we will demonstrate that these structural assumptions have direct implications for individual interest profiles. Specifically, using the SSM (Gurtman & Balakrishnan, 1998), it is possible to reduce observed interest profiles to three theoretically meaningful parameters. Moreover, we will show that the SSM also constitutes a model for assessing correlations between model‐based interest profiles.

### 1.2 A person‐centered approach to interest profile similarity

#### 1.2.1 Structural summary method

Given the validity of the circumplex model, the SSM can be used to fit a cosine function to an individual's observed interest profile. This then results in what we will hereafter refer to as a model‐based interest profile that is defined by three theoretically meaningful parameters. To facilitate the presentation of this approach and the interpretation of these parameters, and to indicate its consequences for analyzing profile similarity, we will use an illustrative example of a fictional mother–son dyad: Sandy and her son, Jeremy, whose model‐based interest profiles are displayed in two alternative representations in Figure 1. For example, Jeremy's interest profile can be modeled with the SSM as:

(1) $$Y_{\mathrm{Jk}}=\tau_{J}+\alpha_{J}\times \cos\left(\theta_{k}-\delta_{J}\right)+d_{k}.$$

In Equation (1), $Y_{\mathrm{Jk}}$ is Jeremy's standardized scale score on interest scale *k*. $\theta_{k}$ indicates the angular position of scale *k* relative to an arbitrary reference category (conventionally, the Realistic scale is chosen as the reference category at 0°). $\tau_{J}$ is Jeremy's profile elevation, that is, the mean of the six interest scores, as represented by the vertical shift of the cosine function (right panel of Figure 1). $\alpha_{J}$ is Jeremy's profile differentiation, an indicator of his intraprofile variability or scatter, as represented by the vector length (left panel) and the amplitude of the cosine function (right panel). $\delta_{J}$ is Jeremy's profile orientation, an indicator of which kinds of activities Jeremy prefers, which is represented by the angular orientation of the vector (left panel) and the angular displacement of the cosine function relative to the reference category (right panel). Lastly, $d_{k}$ is a scale‐specific residual component.

Accordingly, we see that Jeremy has a slightly below average profile $\left(\tau_{J}=-0.35\right)$ that is well differentiated ${(\alpha }_{J}=0.67)$. Furthermore, he is primarily interested in a blend of Realistic and Investigative activities $\left(\delta_{J}=47.3^{\circ }\right)$. Taken together, the SSM provides a parsimonious model for summarizing interest profiles that takes the theoretical implications underlying the vocational interest construct into account. Recent studies have provided empirical support for the importance of explicitly considering these implications in the analysis of associations between vocational interests and external criteria (Nagy et al., 2012; Tracey et al., 2014; Volodina et al., 2015; Warwas, Nagy, Watermann, & Hasselhorn, 2009). A detailed description of how to calculate the model‐based profiles and the SSM parameters from the observed interest profiles is given in the online supplemental materials.

#### 1.2.2 Similarity between model‐based interest profiles

The advantages of the SSM become even more evident when it comes to assessing the similarity between two individuals’ interest profiles. The central aspect of interest profile similarity is the question of how similar individuals’ preferred activities are. The SSM concentrates this information on a single parameter: profile orientation (δ). Consequently, the difference between two individuals’ profile orientations is a natural indicator of the similarity between their model‐based interest profiles. Even more, it holds that the correlation between two model‐based profiles is equal to the cosine of the difference between profile orientations. In this regard, the SSM provides a model for assessing profile correlations derived from theoretically meaningful parameters.

Returning to our illustrative example, we can now compare Sandy's model‐based profile to Jeremy's. In Figure 1, the similarity between the two profiles is indicated by the proximity (i.e., the smallest separating angle) of the person vectors (left panel) and, accordingly, the proximity of the maxima of the cosine functions (right panel). Because Sandy is primarily interested in Social and Enterprising activities $\left(\delta_{S}=202.4^{\circ }\right)$, her interests are very dissimilar to Jeremy's, as indicated by the large difference between their profile orientations $\left(\delta_{S}-\delta_{J}=155.1^{\circ }\right)$, or, equivalently, by the strongly negative profile correlation ${(r}_{\hat{S},\hat{J}}=\cos\left[-155.1^{\circ }\right]=-0.91)$.

Generally, two model‐based interest profiles are most similar when the corresponding vectors point in the same direction (i.e., their separating angle is 0° and their profile correlation equals 1). Likewise, they are most different when the vectors point in opposite directions (i.e., their separating angle is 180° and their profile correlation equals –1). Therefore, a profile correlation of 0 does not mean that two profiles are unrelated, but rather indicates that the respective vectors are 90° apart and, thus, moderately similar.

In sum, the SSM constitutes a model for assessing profile similarity via profile correlations. Moreover, profile correlations between model‐based interest profiles can be straightforwardly interpreted as the differences between profile orientations. Consequently, further support for the validity of the structural model and the SSM would be provided if the profile correlations between model‐based interest profiles were comparable to the correlations between corresponding observed interest profiles.

#### 1.2.3 The problem of gender normativeness

Regardless of whether profile similarity is analyzed on the level of observed or model‐based interest profiles, psychological profiles inevitably contain a normative component (Borkenau & Leising, 2016; Furr, 2008; Wood & Furr, 2016). In the case of vocational interests, normativeness manifests itself in the pronounced differences between women and men (Su, Rounds, & Armstrong, 2009). For example, fathers and sons can be expected to be more similar than fathers and daughters, simply because they are both more likely to score high on male‐typed interest scales (e.g., Realistic and adjacent scales). Consequently, on average, randomly paired same‐sex dyads can be expected to be positively correlated, whereas randomly paired other‐sex dyads can be expected to be negatively correlated. As we will argue in the upcoming sections, neglecting these normative profile components can lead to erroneous conclusions regarding the statistical significance of interest profile similarity of same‐sex and other‐sex dyads.

### 1.3 Interest similarity within families

Theoretically, there are several explanatory approaches from different fields of psychological research to explain why mothers and fathers, on the one hand, and parents and their children, on the other hand, should have similar vocational interests. While it is far beyond the scope of this study to disentangle these mechanisms, we want to briefly address the most plausible explanations before discussing the empirical findings.

#### 1.3.1 Interest similarity between parents

Concerning interest similarity between parents, research on assortative mating and couple similarity suggests at least three plausible mechanisms to explain why partners should have similar interests (Luo, 2017). First, interest similarity could be a result of initial selection, meaning that people choose partners who are psychologically similar to themselves. Second, interest similarity could be due to social homogamy. For example, couples who meet at the workplace or during their studies are likely to have similar interest profiles because such environments typically comprise individuals with similar psychological characteristics (Schneider, 1987). Lastly, vocational interests of parents could be similar because their interests converge over the course of their partnership (Luo & Klohnen, 2005).

Empirically, couple similarity has been found mainly for attitudes, values, and leisure interests, and less for personality traits (e.g., Gonzaga, Carter, & Buckwalter, 2010; Luo & Klohnen, 2005; Watson et al., 2004). However, the question of whether or not vocational interests are a “matchmaker” remains unsettled. The only study so far to analyze interest similarity of mothers and fathers based on Holland's interest typology was conducted by Grotevant, Scarr, and Weinberg (1977). Relying on both VCAs and PCAs, the authors found average profile correlations (APCs) between mothers and fathers to be small, positive, and not significantly different from zero $\left(\overset{¯}{r}=0.11\right)$. However, as argued before, one would expect the APCs of randomly paired women and men to be negative, due to gender normativeness. Bearing this in mind, the results of Grotevant et al. (1977) might in fact indicate meaningful profile similarity between mothers and fathers.

#### 1.3.2 Interest similarity between parents and children

Regarding parent–child interest similarity, there are, again, at least three plausible explanations for why their interests should be similar. First, parents shape the child‐rearing environment to fit their own interests, thus providing specific environmental opportunities and limitations. The child's interactions with this specific environment, and the people within it, should affect her or his vocational personality. Second, children tend to mimic adults they have either actively chosen as a role model, or for whose imitation they receive positive reinforcement (Bandura, 1986). Because the most plausible role models in childhood and early adolescence are the parents, children are likely to engage in and favor activities similar to those preferred by their parents. Lastly, nearly all psychological characteristics have been found to be heritable to some extent (e.g., Bouchard & McGue, 2003). Several studies show that vocational interests are no exception to this rule (Betsworth et al., 1994; Moloney, Bouchard, & Segal, 1991).

Up to this day, only a few empirical studies have tackled the question of how similar RIASEC interests of parents and their children actually are. Thereby, interest associations have often been examined via VCAs, which fail to assess actual between‐person similarity, or via specific kinds of PCAs that were, however, not optimal for several reasons. For example, Grandy and Stahmann (1974) and DeWinne, Overton, and Schneider (1978) analyzed the correspondence of RIASEC types of parents and their children. In sum, they found correspondence between interest types for almost all parent–child dyads, but especially for father–child dyads. Although this typological approach can be considered person‐centered, it reduces the multivariate profile to a single score (i.e., the highest score of the profile) and discards the remaining profile information. Moreover, vocational interests of parents were inferred from their actual occupations rather than being psychometrically assessed.

Grotevant et al. (1977) also analyzed interest similarity between parents and children in biological and adoptive families. To this end, they employed both VCAs (i.e., correlating interest trait scores of parents and children) and PCAs (i.e., correlating entire interest profiles). The authors found APCs in biological families to be positive for all parent–child dyads, whereas those in adoptive families were not significantly different from zero. However, just because the APC of, for example, mother–son dyads in adoptive families was not significantly different from zero does not automatically imply that the APC of mother–son dyads from adoptive families could not be substantially larger than that of randomly paired mothers and sons. Such a finding would support either of the first two explanations for parent–child similarity stated above.

In a more recent study, Holtrop, Born, and De Vries (2017) found normative and distinctive interest profiles of parent–child dyads to be positively correlated, whereas gender had no impact on parent–child interest similarity. However, parent–child interest similarity was not analyzed separately for same‐sex and other‐sex parent–child dyads. Although the authors considered the normativeness problem by analyzing distinctive profile similarity, they did not calculate these distinctive profiles separately for male and female participants, which would have been necessary in order to thoroughly address gender normativeness.

After reviewing the existing body of literature on interest similarity within couples, on the one hand, and between parents and children, on the other hand, it stands out that the question of whether or not the former could affect the inference about the latter has never been addressed. This is surprising because neglecting mother–father similarity could obscure potentially differential associations of same‐sex and other‐sex parent–child dyads. For example, research has shown that adolescent children identify more with their same‐sex parent than with their other‐sex parent (Starrels, 1994). Consequently, if vocational interest development is at least partially influenced by social learning, this could also affect the inference about the similarity between the child and the same‐sex and other‐sex parent. In order to appropriately identify such differences, mother–father similarity must be accounted for.

### 1.4 The present investigation

In the present study, we addressed three interrelated questions about interest profile similarity within families, while trying to overcome the aforementioned methodological challenges. To this end, we analyzed vocational interests of secondary school students and their parents. The students were at the stage of their education where they had to decide which vocational or higher secondary education program to apply for. This phase is marked by the emerging disengagement from the parents and the search for a (vocational) identity.

The first two questions involved the examination of interest profile similarity between mothers and fathers as well as between parents and children. Results of previous research concerned with these questions have been equivocal and could be challenged for methodological reasons. As argued above, a thorough analysis of interest profile similarities should explicitly consider (a) the entire interest profile, (b) the underlying circular ordering of the scales that define it, and (c) gender normativeness, as well as the challenges these specificities pose for the statistical evaluation of these similarities.

Consequently, we hypothesized that randomly paired same‐sex dyads (fathers and sons, mothers and daughters) would have similar interest profiles, as indicated by positive, nonzero APCs. Furthermore, interest profiles of corresponding dyads within families were expected to be even more similar, resulting in even larger APCs. Likewise, we expected interest profiles of randomly paired other‐sex dyads (mothers and fathers, mothers and sons, fathers and daughters) to be rather dissimilar, as indicated by negative, nonzero APCs. Again, interest profiles of corresponding dyads within families were expected to be more similar, as indicated by larger APCs.

Finally, we examined whether or not accounting for mother–father interest similarity affected the inference about parent–child interest similarity. If, for example, vocational interest profiles of mothers and sons within real families were significantly more similar than those of randomly paired mothers and sons, there could be two alternative explanations for this finding. First, a mechanism of interest development could directly cause mother–son interest similarity (e.g., social learning or genetics). Second, mother–son interest similarity could be caused indirectly because both the interest profiles of fathers and sons (e.g., due to a developmental mechanism) as well as those of mothers and fathers (e.g., due to assortative mating) are similar.

If the first scenario is true, then accounting for mother–father similarity should not affect the APC of the randomly paired mothers and sons at all. However, if the second scenario is true, then accounting for mother–father similarity should systematically affect the APC of the randomly paired mothers and sons. In our study, we approached this question indirectly. That is, we examined whether accounting for mother–father similarity in the random pairing procedure changed the reference APC to such a degree that it affected the inferences drawn. In the absence of a sound empirical basis for favoring either of the two alternative scenarios, we addressed this issue as an open research question.

Throughout our study, we examined the aforementioned questions on the level of observed interest profiles, as well as on the level of model‐based circumplex profiles. Because there is sound empirical evidence for the validity of the circumplex model (e.g., Nagy et al., 2010), we expected the interest similarity indices on both levels of analysis to be very similar. Such a finding would further support the validity of the circumplex model of vocational interests because it would mean that the model‐based profiles capture the essential information contained in the observed profiles.

## 2 METHOD

### 2.1 Participants and procedure

Data were taken from the Transformation of the Secondary School System and Academic Careers ‐ Grade 10 (TOSCA‐10) study in Germany (Trautwein, Neumann, Nagy, Lüdtke, & Maaz, 2010). The full sample consisted of *N*=3,047 tenth graders from secondary schools. Parents were asked to complete a separate questionnaire at home. We excluded participants whose parents did not answer any items on the interest scales, leaving us with a final sample of *N*=1,624.

Differences in the RIASEC scale scores of excluded and included participants were negligible across all scales $(|\hat{d}\leq 0.17,median\left|\hat{d}=0.08\right)$. Students who remained in our sample were slightly younger $\left(\hat{d}=-0.31\right)$, had higher cognitive abilities $\left(\hat{d}=0.39\right)$, and had better grades $\left(0.14\leq \hat{d}\leq 0.27\right)$. The proportion of female participants was 54%. Students’ average age was 16.7years. Analyses were conducted on *N*=1,231 mother–father dyads, *N*=853 mother–daughter dyads, *N*=709 mother–son dyads, *N*=686 father–daughter dyads, and *N*=601 father–son dyads.

### 2.2 Measure

#### 2.2.1 General Interest Structure Test (GIST)

The GIST (Bergmann & Eder, 2005) is an instrument used to assess vocational interests according to Holland's (1997) RIASEC model. There is clear evidence for the structural and external validity of the instrument in Germany (Nagy et al., 2010; Volodina & Nagy, 2016). Participants were asked to rate how much they like certain activities on a 5‐point Likert scale (1=*strongly dislike* to 5=*strongly like*). In this study, a shorter 30‐item version of the GIST was used. Despite the lower item number per scale, the internal consistencies were satisfactory throughout the six scales (Cronbach's α=0.68 to α=0.82 in the subsamples).

### 2.3 Statistical analyses

#### 2.3.1 Circumplex structure

The first step in our analyses was to investigate the structure of interests in our sample. To this end, we used the confirmatory cosine function model (CFM) proposed by Nagy, Marsh, Lüdtke, and Trautwein (2009) in which the covariance between two RIASEC scores $y_{j}$ and $y_{k}$ is modeled as.

(2) $$Cov\left(y_{j},y_{k}\right)=\zeta_{j}\times \left[\beta_{0}+\beta_{1}\times \cos\left(\theta_{j}-\theta_{k}\right)\right]\times \zeta_{k},$$

where $\zeta_{j}$ and $\zeta_{k}$ indicate scale‐specific scaling parameters, $\beta_{0}$ and $\beta_{1}$ are the parameters of the correlation function to be estimated $(\beta_{0}+\beta_{1}=1$, $\beta_{0}>0$, and $\beta_{1}>0$), and $\theta_{j}$ and $\theta_{k}$ are the angular positions of the scales. This model is a special case of the stochastic process model proposed by Browne (1992) and is discussed in detail in Nagy et al. (2009). In the present application, the θ parameters were fixed to resemble a perfectly equidistant circumplex, and the scaling parameters ζ were set to be equal across all scales. Model fit was assessed using Mplus 7.4 software (Muthén & Muthén, 1998–2014) with the TYPE=COMPLEX option to account for clustering in families. Goodness‐of‐fit indices were evaluated against the cut‐off values recommended by Marsh, Hau, and Wen (2004).

#### 2.3.2 Calculation of model‐based profiles

In order to compute the model‐based profiles, the RIASEC scales were standardized using the metric defined by the parent subsample. To account for the different sample sizes of mothers and fathers, we weighted the sample means and standard deviations accordingly. We then used ordinary least squares multiple regressions with the cosine and sine of the theoretical scale positions (0°, 60°, … , 300°) as fixed predictors to calculate the model‐based profile and the SSM parameters for each individual in our sample (Gurtman, 1996). A detailed description of this procedure is presented in the online supplemental materials of this article.

#### 2.3.3 Calculation of interest similarity

While there are several approaches to assess similarity between two psychological profiles (e.g., profile covariances or profile correlations, specific decompositions thereof [Furr, 2008], or measures of spatial profile distance [Cronbach & Gleser, 1953]), the use of profile correlations is an obvious choice when it comes to assessing similarity between vocational interest profiles. The main reason for this is that the SSM provides a model for assessing the correlation between two model‐based interest profiles that is merely a function of the central parameters of the respective interest profiles: the profile orientations. Consequently, for each dyad in our sample, we calculated the Pearson product‐moment correlation between the two standardized observed profiles, as well as that between the corresponding model‐based profiles. As recommended by Silver and Dunlap (1987), the correlations were then transformed to Fisher's *z* scores, averaged across the sample (for each dyad type), and retransformed to the *r* metric afterward.

#### 2.3.4 Evaluation of interest similarity

In order to ensure an adequate evaluation of the statistical significance of these profile similarity indices, we relied on a so‐called pseudo‐couple approach (Furr, 2008; Kenny, Kashy, & Cook, 2006). This was necessary because we expected the APCs of randomly paired family members to be different from zero due to gender normativeness. Following this approach, a suitable reference distribution is found by creating a sufficient number (in our case, 1,000) of randomly reorganized pseudo‐family data sets. Within each pseudo‐family data set, the children were randomly paired with a new mother and a new father, each from a different dyad than in the real‐family data set. Furthermore, the randomization was restricted to the extent that every daughter (or son) was matched with a random mother and a random father, who also had a daughter (or a son) in the real‐family data set, to control for possible influences of the child's gender on parent–child similarity.

We then calculated the APCs for each dyad type in each of the 1,000 pseudo‐family data sets, thus obtaining empirical distributions for these similarity measures. The means of these empirical distributions constitute suitable reference values that implicitly take gender normativeness into account. For example, if randomly paired mothers and sons truly had, on average, rather dissimilar profiles, the respective empirical distribution would be centered around a negative value. Moreover, the 0.5% and 99.5% quantiles of the empirical distributions serve as the upper and lower boundaries of 99% confidence regions for these APCs. Consequently, if an APC from a real‐family dyad fell outside this region (i.e., if it were larger than the upper limit of this interval), we would conclude that the interest profiles for this specific dyad were substantially more similar than would be expected by chance.

Finally, in order to account for the similarity between the parents’ interest profiles, we proceeded almost exactly as described above, while placing further constraints on the resampling process. Specifically, we split the data across different levels of mother–father profile similarity and repeated the resampling procedure within these intervals. For example, for mother–child similarity, the data sets were split across different levels of profile similarity between the real fathers and mothers. Consequently, the randomly assigned mothers were roughly as similar to the real fathers as were the real mothers. We thus obtained reference distributions of mother–child APCs, conditional on mother–father profile similarity. Mother–father dyads with extreme profile similarities were excluded because there were too few cases to resample in these groups (N=37; this affected mother–father dyads with profile covariances smaller than –0.6 or larger than 0.8).

The primary advantage of this technically demanding approach is that it allows accounting for both gender normativeness and mother–father similarity indirectly, while the meaning of the (average) profile correlation indices remains unchanged. Consequently, the possibly confounding effects of these factors only influence the decision about the statistical significance of the APCs, and not the real‐family APCs themselves. A formal description of this rationale is presented in the online supplemental materials. All analyses described in this section were conducted using self‐written functions in R (version 3.3.1; R Core Team, 2016).

## 3 RESULTS

### 3.1 Circumplex structure

The first step in our analyses was to evaluate the structural validity of the circumplex model in our sample. Table 1 shows the correlation matrix of the RIASEC scales in the full sample. The intercorrelations closely resembled the typical pattern implied by the circumplex structure (e.g., Gurtman & Balakrishnan, 1998). That is, for each scale, there was a pattern of decreasing correlations mirroring the increasing distance of the scales on the circumplex. Passing the scales that are on the opposite side of the circumplex, the correlations increased again. In order to assess the fit of the model‐implied correlation pattern, the cosine function model (CFM; Nagy et al., 2009) was fitted to the data. Despite the highly specific constraints of the CFM (i.e., fixed scale positions and equal scaling factors), model fit was satisfactory, $\chi^{2}\left(13,N=4,545\right)=428.25,\;\;p=0.00,\;CFI=0.924,\;\;RMSEA=0.084$, thus supporting the validity of the postulated circumplex structure.

**Table 1:** Correlation matrix of RIASEC scales in the full sample

	R	I	A	S	E	C
R	(0.79)					
I	0.38	(0.75)				
A	0.08	0.51	(0.74)			
S	–0.03	0.26	0.45	(0.79)		
E	0.19	0.31	0.34	0.49	(0.77)	
C	0.29	0.28	0.18	0.31	0.51	(0.77)

Notes

R=Realistic; I=Investigative; A=Artistic; S=Social; E=Enterprising; C=Conventional. Internal consistencies (Cronbach's *α*) of the scales in the full sample are reported in parentheses.

**Figure 2:**
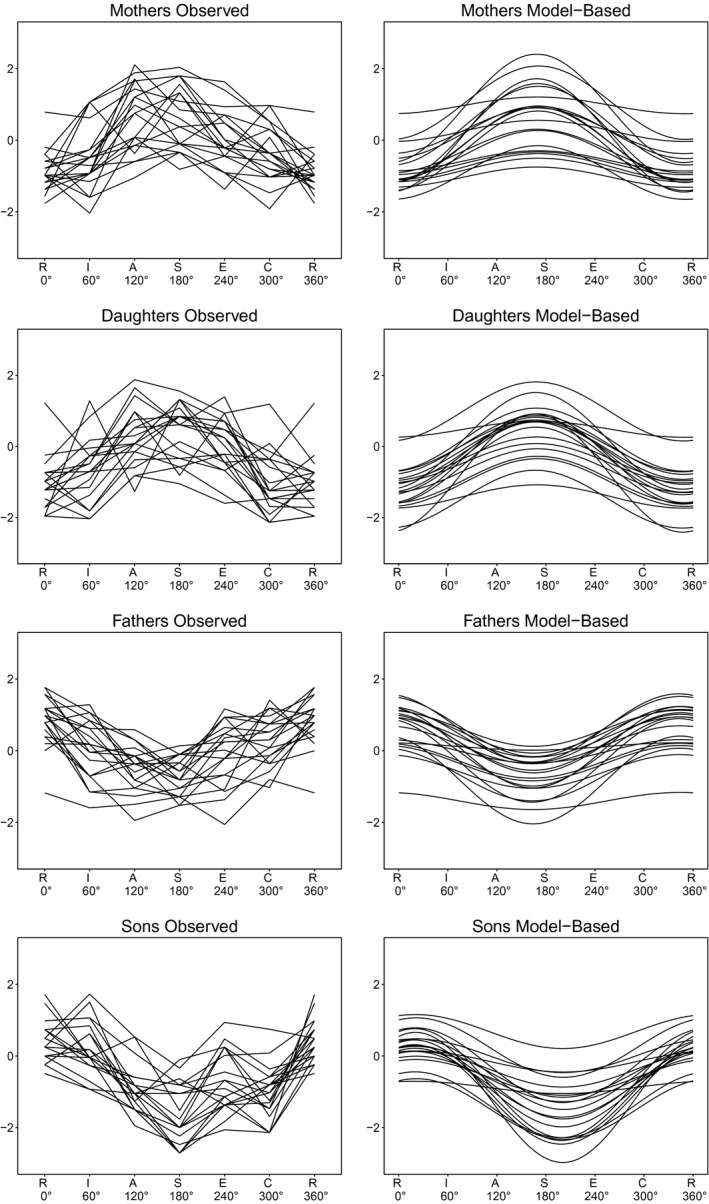
Line plots for prototypical mother, father, daughter, and son profiles. *Notes*. Left side: standardized observed profiles; right side: model‐based profiles. R=Realistic; I=Investigative; A=Artistic; S=Social; E=Enterprising; C=Conventional

As argued above, the validity of the circumplex structure has direct implications for individual interest profiles. That is, it warrants the application of the SSM to obtain model‐based interest profiles. To visualize the appropriateness of this approach, Figure 2 displays the observed (left side) and the model‐based (right side) profiles of 20 individuals from each group (mothers, fathers, sons, and daughters). As can be seen, the model‐based profiles closely resembled the observed profiles, especially with regard to central characteristics (e.g., profile shape and scatter). Correlations between model‐based and observed profiles across all participants were very high $\left(\overset{¯}{r}=0.79\right)$. In Figure 2, we specifically chose individuals who had profile orientations close to the respective profile orientation group mean, in order to demonstrate the importance of the gender normativeness problem. Clearly, the prototypical profiles of same‐sex individuals were very much alike, whereas those of other‐sex individuals were highly dissimilar. The descriptive statistics of the SSM parameters for each group can be found in the online supplemental materials.

### 3.2 Univariate scalewise associations

We first examined the bivariate correlations between corresponding observed RIASEC scores (first supercolumn of Table 2). Interest scale scores of mothers and fathers were highly correlated (0.35≤r≤0.56), whereas those of parents and children were, if at all, only weakly correlated (0.00≤r≤0.25). However, because this procedure corresponds to a VCA, these results do not convey information about the similarity between the interest profiles of the respective dyads. Furthermore, these bivariate correlations confound associations between theoretically meaningful profile components (e.g., profile orientation) with those attributable to less important aspects of the profile (e.g., profile elevation).

**Table 2:** Correlations between observed scale scores and SSM parameters

Dyad type	Scale scores						SSM parameters		
	R	I	A	S	E	C	*τ*	*α*	$\overset{-}{\Delta_{\delta }}$
Mother–father	0.36*	0.56*	0.53*	0.52*	0.55*	0.38*	0.68*	0.13*	82.7°
Mother–son	0.10*	0.12*	0.10*	0.00	0.05	0.08*	0.01	0.12*	89.1°
Mother–daughter	0.16*	0.19*	0.25*	0.14*	0.13*	0.10*	0.15*	0.15*	59.3°
Father–son	0.21*	0.22*	0.14*	0.09*	0.02	0.08	0.05	0.13*	71.2°
Father–daughter	0.21*	0.19*	0.20*	0.18*	0.16*	0.01	0.19*	0.02	102.9°

Notes

R=Realistic; I=Investigative; A=Artistic; S=Social; E=Enterprising; C=Conventional; SSM=structural summary method; *τ*=profile elevation; *α*=profile differentiation; $\overset{-}{\Delta_{\delta }}$ = angular mean of difference between profile orientations.

* *p* < 0.05.

To address this problem, we display the associations between the respective SSM parameters in the second supercolumn of Table 2. For mothers and fathers, we found that a large part of the scale‐level associations was attributable to the high correlation between profile mean levels (τ;r=0.68). Corresponding correlations between parent–child dyads were small or not significant (0.01≤r≤0.19). Regarding profile differentiation (α), correlations between all dyad types were small or nonsignificant (0.02≤r≤0.15). Lastly, the angular means of the differences in profile orientations (scaled from 0° to 180°) were smaller for same‐sex dyads $\left(59.3^{\circ }\leq \overset{¯}{\Delta_{\delta }}\leq 71.2^{\circ }\right)$ than for other‐sex dyads $\left(82.7^{\circ }\leq \overset{¯}{\Delta_{\delta }}\leq 102.9^{\circ }\right)$. Taken together, these results suggest that there are meaningful associations between interest profiles within families and emphasize the need to take the entire profile into account.

### 3.3 Interest profile similarity

The next step aimed to overcome the abovementioned limitations of VCAs by moving on to the analyses of interest profiles. The upper part of Table 3 presents the APCs for the real‐family dyads, as well as the means and the limits of the interval containing 99% of the corresponding APCs from the resampling distributions for both the observed profiles (left) and the model‐based profiles (right). As expected, randomly paired same‐sex dyads yielded substantially positive APCs, whereas randomly paired other‐sex dyads yielded substantially negative APCs. Moreover, the APCs in the real‐family data set were all significantly larger than those obtained for the pseudo‐families.

**Table 3:** Average profile correlations for observed and model‐based profiles

Dyad type	Observed profiles			Model‐based profiles			
	$\overset{¯}{r}$	${\overset{¯}{r}}^{pseudo}$	99% PL	$\overset{¯}{r}$	${\overset{¯}{r}}^{pseudo}$	99% PL	
*Before accounting for mother–father similarity*						
Mother–father	0.18	–0.18	[–0.22, –0.14]	0.17	–0.34	[–0.43, –0.26]
Mother–son	–0.02	–0.15	[–0.19, –0.10]	0.02	–0.20	[–0.33, –0.09]
Mother–daughter	0.35	0.19	[0.15, 0.23]	0.62	0.45	[0.36, 0.54]
Father–son	0.29	0.11	[0.06, 0.17]	0.44	0.19	[0.05, 0.33]
Father–daughter	–0.07	–0.21	[–0.25, –0.16]	–0.32	–0.50	[–0.58, –0.40]
*After accounting for mother–father similarity*						
Mother–son	–0.04	–0.08	[–0.16, –0.02]	–0.03	–0.11	[–0.27, 0.07]
Mother–daughter	0.34	0.19	[0.13, 0.25]	0.60	0.42	[0.29, 0.54]
Father–son	0.30	0.13	[0.04, 0.20]	0.46	0.22	[0.04, 0.37]
Father–daughter	–0.08	–0.13	[–0.19, –0.07]	–0.34	–0.37	[–0.48, –0.25]

Notes

$\overset{¯}{r}$ = average profile correlations for the real families; ${\overset{¯}{r}}^{pseudo}$ = average profile correlations (APCs) for the pseudo‐families; 99% PL=lower (0.005 percentile) and upper (0.995 percentile) limits of the empirical distributions for the respective pseudo‐family APCs. APCs from real families are considered statistically significant if the estimate is outside of the boundaries of the corresponding 99% PL.

#### 3.3.1 Observed profiles

For mother–father dyads, the observed APC was ${\overset{¯}{r}}_{M,F}=0.18$, whereas random pairing yielded an APC of ${\overset{¯}{r}}_{M,F}^{pseudo}=-0.18$. Although APCs of other‐sex parent–child dyads found in the real‐family data set were close to zero and negative $({\overset{¯}{r}}_{M,S}=-0.02$, and ${\overset{¯}{r}}_{F,D}=-0.07)$, they were still significantly larger than would be expected by chance $({\overset{¯}{r}}_{M,F}^{pseudo}=-0.15$, and ${\overset{¯}{r}}_{F,D}^{pseudo}=-0.21)$. For same‐sex dyads, APCs of pseudo‐families were positive and of moderate size $({\overset{¯}{r}}_{M,D}^{pseudo}=0.19$, and ${\overset{¯}{r}}_{F,S}^{pseudo}=0.11)$. However, APCs of real families were significantly larger $({\overset{¯}{r}}_{M,D}=0.35$, and ${\overset{¯}{r}}_{F,S}=0.29)$.

Taken together, our findings were fully in line with our expectations. Furthermore, they underline the need to consider gender normativeness when evaluating the statistical significance of interest profile similarities. The largest difference between APCs of real and randomly paired dyads was found for mothers and fathers $\left(\Delta \overset{¯}{r}=0.36\right)$. The differences between APCs of real and randomly paired parent–child dyads were smaller and of comparable size $\left(0.13\leq \Delta \overset{¯}{r}\leq 0.18\right)$.

#### 3.3.2 Model‐based profiles

The findings for the model‐based interest profiles (upper right part of Table 3) were consistent with those on the level of observed profiles. The absolute values of the model‐based APCs were somewhat larger than those of the observed APCs. However, this can be explained by the variance reduction that goes along with the transition from the observed to the model‐based profiles (comparable to the variance reduction in latent variable modeling, where variance caused by measurement error is removed). If the covariances stay roughly the same and the variances decrease, the correlations inevitably become larger. Indeed, when we looked at the average profile covariances, we did not find such pronounced differences.

As argued above, correlations between model‐based profiles are a function of the difference between profile orientations. We thus provide the angular means of the smallest separating angles between profile orientations for all dyad types in the rightmost column of Table 2. The angular means of real‐family dyads were significantly smaller (i.e., the profiles were significantly more similar) than the respective angular means from the resampling distributions. A graphical approach that facilitates a better understanding of the differences in profile orientations between real families and pseudo‐families is presented in the online supplemental materials.

In summary, our findings further underline the validity of the circumplex model of vocational interests. Specifically, they confirm that the model‐based profiles contain the essential information about the raw interest profiles. This argument is further supported by the high correlations between the observed and the model‐based profile similarities (by means of profile covariances) across all dyad types $\left(0.85\leq r_{obs,mod}\leq 0.92\right)$.

#### 3.3.3 Accounting for parent similarity

The final set of analyses tackled the question of whether or not the inference about interest similarity between parents and their children was affected by the interest similarity between mothers and fathers. We argued that if parent–child similarity was caused by a dyad‐specific developmental mechanism, accounting for mother–father similarity should not affect the inference about parent–child similarity (see The Present Investigation section). Although such a finding would not be sufficient to prove the existence of a causal mechanism, it would be a sine qua non that would pave the way for subsequent research on cause and effect of parent–child interest similarity.

The lower part of Table 3 presents the results for every parent–child dyad type on the level of observed (left side) and model‐based profiles (right side), after accounting for mother–father similarity. APCs of same‐sex parent–child dyads from real families $({\overset{¯}{r}}_{M,D}=0.34$, and ${\overset{¯}{r}}_{F,S}=0.30)$ were, again, significantly larger than those from pseudo‐families $({\overset{¯}{r}}_{M,D}^{pseudo}=0.19$, and ${\overset{¯}{r}}_{F,S}^{pseudo}=0.13)$. However, APCs of other‐sex parent–child dyads from real families were negative $({\overset{¯}{r}}_{M,S}=-0.04$, and ${\overset{¯}{r}}_{F,D}=-0.08)$ and no longer significantly different from those obtained in pseudo‐families $({\overset{¯}{r}}_{M,S}^{pseudo}=-0.08$, and ${\overset{¯}{r}}_{F,D}^{pseudo}=-0.13)$. Again, the results were basically identical for the model‐based profiles. Taken together, our findings indicate that only same‐sex parent–child dyads are, on average, more similar to each other than would be expected by chance when mother–father similarity is accounted for. Consequently, interest similarity between other‐sex parent–child dyads appears to be traceable to the similarity between the interests of mothers and fathers.

## 4 DISCUSSION

The research presented in this article aimed to extend the understanding of intrafamily similarities in vocational interests. To this end, we identified two major methodological limitations of previous research on this topic and demonstrated the challenges they pose for analyzing profile similarity. We then combined well‐established methodological means to overcome them. Specifically, we used the SSM (Gurtman & Balakrishnan, 1998) to explicitly consider the circumplex structure underlying the vocational interest scales. In doing so, we were able to assess interest profile similarity as a function of the profiles’ central parameter: profile orientation. Furthermore, we used a person‐centered pseudo‐couple approach to adequately account for systematic differences between interests of women and men, thus taking the gender normativeness problem into account. In sum, our findings suggest that mothers and fathers, on the one hand, and parents and their children, on the other hand, have substantially similar interest profiles. However, we also found indications that the reasons for these similarities might be different for same‐sex and other‐sex parent–child dyads.

### 4.1 Interest similarity between parents

The first major finding of our study was that interest profiles of mothers and fathers are substantially more similar than those of randomly paired women and men. Moreover, mother–father similarity was noticeably larger than parent–child similarity. Due to the cross‐sectional research design, we could not disentangle whether these similarities were due to assortative mating or were a result of couples becoming more similar over time. However, several studies that analyzed couple similarity with conceptually related constructs suggest that couple similarity is stable (Caspi, Herbener, & Ozer, 1992) and that couples are similar even before they meet (Gonzaga et al., 2010). Consequently, we suspect that mother–father interest similarity is due to active selection or social homogamy, rather than convergence. However, more research is needed to ultimately decipher these mechanisms.

### 4.2 Interest similarity between parents and children

For parent–child interest similarity, the moderately sized positive correlations between corresponding RIASEC scale scores were comparable to those of parent–child personality trait similarity (Prinzie et al., 2004). Interest profiles of all parent–child dyads were more similar than those of corresponding randomly paired parents and children. However, the finding that only same‐sex parent–child dyads were significantly more similar to each other when mother–father similarity was accounted for suggests that the reasons for why these similarities occur might be different for same‐sex and other‐sex parent–child dyads.

One possible explanation could be that children's vocational interest development is substantially shaped by social learning (e.g., Lent et al., 1994) and that the same‐sex parent is the primary source of identification. Support for this assumption can be found in the work of Starrels (1994), who demonstrated that adolescent children identify more strongly with their same‐sex parent. Moreover, Holtrop et al. (2017) showed that children, when asked to choose a parent whose interests they were supposed to rate, tend to select the same‐sex parent rather than the other‐sex parent. Thus, there is some empirical support for differences in identification with the same‐sex and the other‐sex parent, but further examinations of the associations between identification and interest development were beyond the scope of our study. Future research could pursue longitudinal research designs that also incorporate measures of parental identification to address this research gap.

At this point, we want to stress that the similarities reported here are average profile similarities. Consequently, we do not wish to claim that the other‐sex parent could not possibly influence the development of the same‐sex child's vocational interests. In our analyses, we found considerable variability in interest similarities. That is, there were families in which sons and daughters were very similar to their other‐sex parent and not so much to their same‐sex parent. Future research could investigate the mechanisms underlying this variability more thoroughly by identifying moderators of parent–child interest similarity.

### 4.3 Methodological advances and opportunities

From a methodological point of view, our results provide additional support for the validity of the circumplex structure underlying Holland's (1997) model of vocational interests. That is, we were able to show that the model‐based interest profiles, as calculated via the SSM, capture the essential information contained in the observed profiles. This conclusion is supported by the strong correspondence between observed and model‐based interest profiles, on the one hand, and the strong concordance between the similarities calculated from observed and model‐based interest profiles, on the other hand.

Furthermore, adopting the perspective of circular interest profiles enabled us to identify crucial methodological challenges that severely limit the validity of previous findings on interest profile similarity. Specifically, the typical assumption that randomly pairing individuals would result in a zero correlation between the characteristics in question is erroneous in the case of vocational interest profile similarity, where, due to the pronounced gender differences (Su et al., 2009), high similarity (dissimilarity) and, thus, a high (low) APC can be expected even between randomly paired same‐sex (other‐sex) dyads.

Most importantly, the features outlined above are not limited to the analysis of similarity between vocational interest profiles. Instead, they are relevant whenever dyadic associations between psychological constructs that exhibit a circumplex structure, for example, interpersonal behavior (Wiggins, 1979) or affect (Russell, 1980), are examined and whenever the two subsamples that define the dyad differ systematically with regard to profile orientation. For example, Gurtman and Lee (2009) reviewed sex differences in interpersonal behavior and found systematic differences between women and men. Consequently, researchers concerned with the analysis of the similarity between profiles of interpersonal behavior should be wary of similarity (dissimilarity) that is merely due to individuals’ having the same (a different) gender.

## 5 LIMITATIONS AND FUTURE DIRECTIONS

The sample used in this study was selective to the extent that it was limited to German secondary school students and their parents. Furthermore, the sample was positively selected because parents of higher achieving and more intelligent students were more likely to fill out the parent questionnaire. In order to generalize our findings, more data from families from different countries with different cultural, social, and educational backgrounds would be required. Moreover, it would be interesting to analyze parent–child interest similarity with children in different developmental phases.

Finally, our data were collected at one point in time, limiting the scope of our analyses to cross‐sectional associations. Although vocational interests are considered to be relatively stable across the life span (Low et al., 2005), they are—just like personality traits—susceptible to change (Xu & Tracey, 2016). This is especially true for early adulthood and adolescence, where children are still in search of a (vocational) identity. Thus, to gain further insights into the mechanisms underlying interest development, it would be necessary to analyze the covariation of interest profile trajectories between parents and their children across time. Whether interest profile similarity of parents and their children becomes larger, becomes smaller, or does not change at all from early childhood to late adolescence is an interesting question that has not been addressed thus far.

## 6 CONCLUSIONS

Taken together, our study introduces a novel methodological approach for examining interest profile similarity. By combining the SSM (Gurtman & Balakrishnan, 1998) and the pseudo‐couple technique (Kenny et al., 2006), we were able to assess interest similarity directly as the correspondence between preferred activities and to evaluate these similarities with adequate reference distributions that indirectly account for gender normativeness and mother–father similarity. Using this approach, we were able to show that there are substantial similarities between vocational interest profiles of mothers and fathers, and between those of all parent–child dyads. Our findings suggest that interest profile similarity between same‐sex parent–child dyads could be due to specific developmental processes, whereas the similarity between other‐sex parent–child dyads might be attributable to the similarity between the parents.

## CONFLICT OF INTERESTS

The author(s) declared no potential conflicts of interest with respect to the research, authorship, and/or publication of this article.
