# Supplementary material for: Similarity of vocational interest profiles within families: A person‐centered approach for examining associations between circumplex profiles
Source: J Pers. 2018 Aug 30;87(3):593–606. doi: 10.1111/jopy.12418 (PMC7379732; doi:10.1111/jopy.12418)
Supplement: Supplementary file 1 [file JOPY-87-593-s001.docx]

Online Supplemental Materials to

Similarity of Vocational Interest Profiles within Families: A Person-Centered Approach for Examining Associations between Circumplex Profiles

Julian M. Etzel^1^, Oliver Lüdtke^1,2^, Jenny Wagner^1,3^, and Gabriel Nagy^1^

^1^ Leibniz Institute for Science and Mathematics Education, Kiel, Germany

^2^ Centre for International Student Assessment, Germany

^3^ University of Hamburg, Hamburg, Germany

Author Note

Correspondence concerning the online supplemental materials should be addressed to Julian M. Etzel, Olshausenstraße 62, 24118, Kiel, Germany. E-mail: etzel@ipn.uni-kiel.de

**Calculation of Structural Summary Method Parameters**

In this paragraph, we explicate the computation of the model-based interest profiles by means of applying the structural summary method (SSM) to the standardized observed scale scores (Gurtman & Balakrishnan, 1998). Therefore, let *i* be the index for persons and *j* the index for the scales (R, I, A, etc.). Furthermore, let $\theta_{j}$ be the angular position of scale *j* on the circumplex, with Realistic as reference scale in the three o'clock position $(\theta_{R}=0^{\circ})$. The model-based profile scores $\hat{y}_{ij}$are then modeled as:

| $\hat{y}_{ij}=\tau_{i}+{\cos\left( \theta_{j} \right)\times\mathrm{PT}_{i}+\sin\left( \theta_{j} \right)\times DI_{i}=\tau_{i}+\alpha}_{i}\times\cos(\theta_{j}-\delta_{i})$ | (1) |
| --- | --- |

Consequently, $\hat{y}_{ij}$ can be expressed as a function of the profile mean level $(\tau_{i})$ and either 1) the scores on the two circumplex axes People-Things ${(PT}_{i})$ and Data-Ideas ${(DI}_{i}$, see also the next section$)$ weighted by the cosine and sine of the angular position of scale *j* on the circumplex $(\theta_{R}=0^{\circ}, \theta_{I}=60^{\circ}, \theta_{A}=120^{\circ}$, etc.$)$, or, equivalently, 2) profile differentiation $(\alpha_{i})$ and profile orientation ${(\delta}_{i})$. In either notation, the complexity of the interest profile is reduced to three person parameters. The two notations can be converted into each other using the following formulae and the respective inverse functions:

| $\delta_{i}=arctan2\left( \frac{DI_{i}}{PT_{i}} \right) \times\frac{180}{\pi}$ | (2) |
| --- | --- |
|  |  |
| $\alpha_{i}=\sqrt{\mathrm{PT}_{i}^{2}+DI_{i}^{2}}$ | (3) |

In order to obtain the parameters from the standardized observed scale scores, an ordinary least squares (OLS) multiple linear regression is fit for every person. Specifically, the vector of standardized observed scale scores $y_{ij}$ is used as the dependent variable, while the cosine and sine of the scale positions on the circumplex ($\theta_{j}$) are used as two fixed predictors. The intercept of this regression is equal to the profile elevation ($\tau_{i}$), while the two regression weights are equal to the axes-scores ($\mathrm{PT}_{i}$ and $\mathrm{DI}_{i}$). These can then be transformed to profile orientation ${(\delta}_{i})$. and profile differentiation $(\alpha_{i})$, using the formulae presented above. In Table 1, we display the descriptives of the SSM parameters for each group in our sample.

Naturally, individual profiles will vary in their degree of fit to the proposed cosine function. In the SSM, the goodness-of-fit is indicated by the $R^{2}$ measure of the respective OLS regression (Gurtman & Pincus, 2003). Although higher degrees of misfit (a common cutoff is $R^{2}<.70$) are said to result in unstable estimates of the underlying parameters (Wright, Pincus, Conroy, & Hilsenroth, 2009), the extent to which model-based profiles with less optimal fit are nevertheless able to capture the essence of the observed profiles is unclear. In our study, we found that the similarities calculated from observed and model-predicted profiles still correlated highly when only dyads with model-based profiles that did not show a very good fit to the cosine function (i.e., both $R^{2}<.70$) were considered. This means that although a suboptimal fit to the sinusodial form might reduce the reliability of the SSM parameters (Wright et al., 2009) they are still valid to the extent that they capture the essential profile information.

**Dimensions Underlying the Interest Circumplex**

As an additional note to the structural model, we want to add that there is strong consent that the underlying two-dimensional structure, that is spanned by the orthogonal axes People-Things and Data-Ideas, is the core element of the interest circumplex (Prediger, 1982). The People-Things axis is spanned by the Social and Realistic scales, indicating a preference for interpersonal versus impersonal activities. The Data-Ideas axis is orthogonal to the People-Things axis and differentiates between preferences for highly structured, data-driven tasks versus unsystematic tasks that involve theorizing, creativity, and abstraction. Consequently, the six Holland types can be interpreted as different blends of the axes’ poles. Moreover, as shown in Equation 1, the SSM can be used to obtain individuals' scores on these two dimensions.

Because the analyses in our study focused on the, in our opinion, theoretically more meaningful transformations of these dimensional scores (i.e., profile differentiation $\alpha$ and profile orientation $\delta$), we did not report the associations between the intrafamily dyads' axes scores in the main manuscript. Nevertheless, for the sake of completeness, we present the respective correlations in Table 2. As can be seen, both People-Things scores as well as Data-Ideas scores were positively correlated for all dyads types. In line with our other findings, these correlations were largest for mother-father dyads and somewhat smaller, but of comparable size, for all parent-child dyads.

**Statistical Evaluation of Profile Similarity**

The following paragraphs are intended to provide a more formal demonstration of our methodological rationale for evaluating the statistical significance of average profile correlations (APCs). Furthermore, we will elaborate on the approach we used to analyze parent-child interest similarity while accounting for the similarity between interest profiles of mothers and fathers. To this end, we will exemplarily demonstrate our approach for mother-son dyads. Of course, the rationale is identical for all other dyad types. Hereafter, mothers are denoted as *M* and sons as *S*.

The hypotheses analyzed in the first analyses were:

$H_{0}$: The average profile similarity between mothers and sons within real families is equal to that of corresponding dyads from randomly paired families.

$H_{1}$: The average profile similarity between mothers and sons within real families is not equal to that of the corresponding dyads from randomly paired families.

These translate into the following statistical hypotheses:

$H_{0}$: $E\left( r_{M,S} \right)=E\left( r_{M,S}^{\mathrm{pseudo}} \right)$

$H_{1}$: $E\left( r_{M,S} \right)\neq E\left( r_{M,S}^{\mathrm{pseudo}} \right)$

Using the pseudo-coupling approach, we obtained empirical distributions of the APCs across the 1,000 resampling datasets for each of the five dyad types (mother-father, mother-son, etc.). The decision concerning whether or not to reject the respective $H_{0}$ was based on the following rule: Determine the 0.5% and 99.5% quantiles of the empirical distributions of the average similarity measures across the 1,000 pseudo-family datasets. Reject the respective $H_{0}$ if the corresponding APC from the real family dataset falls outside the limits of this confidence region.

In the second set of analyses, we intended to account for the similarity between parents' interest profiles. Specifically, we considered mother-father similarity in our resampling procedure, so that the APCs from the real-family dyads were identical (and thus comparable) to those obtained in the previous analyses. Here, we additionally denote fathers as *F*. Consequently, the statistical hypotheses are as follows:

$H_{0}$: $E\left( r_{M,S} \right)=E\left( r_{M,S}^{\mathrm{pseudo}} \right)| f(\mathrm{Cov}_{M,F})$

$H_{1}$: $E\left( r_{M,S} \right)\neq E\left( r_{M,S}^{\mathrm{pseudo}} \right)| f(\mathrm{Cov}_{M,F})$

That is, we evaluated the average profile similarity of mothers and sons, conditional on the similarity between the interest profiles of the parents. In order to obtain an appropriate estimate for $E\left( r_{M,S}^{\mathrm{pseudo}} \right)| f(\mathrm{Cov}_{M,F})$, we approximated the continuous distribution $f(\mathrm{Cov}_{M,F})$ by a discrete histogram distribution. Therefore, the distribution of mother-father profile covariances was split into *K* bins of equal width (in our case, a bin width of 0.1). We used the profile covariances and not the profile correlations because, in the case of model-based profiles, the latter are slightly inflated due to the variance reduction. The relative bin occupancy in bin *k* is written as $\pi_{k}$ ($\sum_{k=1}^{K} \pi_{k}=1$).

In the next step, we calculated the mother-son APCs within each of the *K* bins (${\bar{r_{k}}}_{M,S}^{\mathrm{pseudo}}$). The final step was to estimate $E\left( r_{M,S}^{\mathrm{pseudo}} \right)| f(\mathrm{Cov}_{M,F})$ by averaging the bin-wise APCs (${\bar{r_{k}}}_{M,S}^{\mathrm{pseudo}}$) from the previous step, while using the relative bin occupancy $\pi_{k}$ obtained in the first step as weights:

| $E\left( r_{M,S}^{\mathrm{pseudo}} \right)\vert f(\mathrm{Cov}_{M,F})\approx\sum_{k=1}^{K} \pi_{k}\times{r_{k}}_{M,S}^{\mathrm{pseudo}}$ | (4) |
| --- | --- |

This approach was repeated for each of the 1,000 datasets. We thus obtained an empirical distribution for the APC of the randomly paired mother-son dyads, while taking mother-father similarity into account. Just like in the previous analyses, the respective APC from the real family dataset was considered significantly different from what would be expected by chance, if it fell outside the 99% confidence region.

**Illustration of Differences in Profile Orientation**

A key strength of the SSM is the possibility to express profile similarity in terms of differences in profile orientations. In other words, profile similarity can be assessed by the smallest separating angle between the two person vectors in the circumplex projection. In our analyses, the angular means of the absolute separating angles (from 0° to 180°) in the real-family data were $\bar{\delta}_{M-F}=82.71^{\circ}$ for mothers and fathers, $\bar{\delta}_{M-D}=59.33^{\circ}$ and $\bar{\delta}_{F-S}=71.18$ for same-sex dyads, and $\bar{\delta}_{M-S}=89.10^{\circ}$ and $\bar{\delta}_{F-D}=102.92^{\circ}$ for other-sex dyads. The corresponding angular means from the randomly paired dyads were roughly 10° larger for all parent-child dyads, and roughly 20° larger for mother-father dyads.

In order to facilitate an evaluation of the differences between the profile orientations of real and pseudo-families, Figure 1 visualizes the smoothed relative frequency distributions of separating angles for the real-family data (black lines) and across the 1,000 pseudo-family datasets (gray lines). For same-sex dyads, the mass of the distributions could be expected to be centered near the 0° position and to decrease towards the outer limits at -180° and 180° (as indicated by the gray lines in the left panels of Figure 1). For other-sex dyads, the minima of the distributions could be expected to be located near the 0° position, and to increase towards the outer limits (as indicated by the gray lines in the right panels of Figure 1). In other words, members of same-sex dyads could be expected to be rather similar than dissimilar, and those of other-sex dyads to be rather dissimilar than similar, due to gender-normativeness.

Comparing the resampling distributions to those obtained from the real-family data, there were two intersections that divided the distributions into specific sections. In between the intersections, the relative frequency of separating angles in the real-family data exceeded that obtained from the pseudo-families. That is, within (or outside) the limits of these intervals, real-family dyads were more similar (or less dissimilar) than corresponding pseudo-couples. For same-sex dyads, this area was narrower (roughly ± 60°) than for other-sex dyads. For the latter, the intervals were wider (roughly ± 70° up to ± 100°). This is because same-sex parent-child dyads are inevitably more similar to each other than other-sex parent-child dyads, due to gender-normativeness. Again, the differences between real-family and pseudo-family dyads were strongest for mother-father dyads.

References

Gurtman, M. B., & Balakrishnan, J. D. (1998). Circular measurement redux: The analysis and interpretation of interpersonal circle profiles. *Clinical Psychology: Science and Practice*, *5* , 344–360. doi: 10.1111/j.1468-2850.1998.tb00154.x

Gurtman, M. B., & Pincus, A. L. (2003). The circumplex model: Methods and research applications. In J. A. Schinka & W. F. Velicer (Eds.), *Handbook of psychology: Research methods in psychology* (pp. 407–428). doi: 10.1002/0471264385.wei0216

Mardia, K. V., & Jupp, P. (2000). *Directional statistics.* Chichester, UK: John Wiley and Sons Ltd.

Prediger, D. J. (1982). Dimensions underlying Holland’s hexagon: Missing link between interests and occupations? *Journal of Vocational Behavior*, *21*, 259–287. doi: 10.1016/0001-8791(82)90036-7

Wright, A. G. C., Pincus, A. L., Conroy, D. E., & Hilsenroth, M. J. (2009). Integrating methods to optimize circumplex description and comparison of groups. *Journal of personality assessment*, *91* , 311–322. doi: 10.1080/00223890902935696

Supplemental Table 1

*Sample Descriptive Statistics of Axes Scores and SSM Parameters*

|  | P-T | |  | D-I | |  | τ | |  | α | |  | δ | |
| --- | --- | --- | --- | --- | --- | --- | --- | --- | --- | --- | --- | --- | --- | --- |
|  | M | SD |  | M | SD |  | M | SD |  | M | SD |  | M | SD |
| Mothers | -0.34 | 0.46 |  | 0.09 | 0.54 |  | -0.04 | 0.66 |  | 0.69 | 0.39 |  | 169.8° | 66.8° |
| Fathers | 0.33 | 0.50 |  | -0.09 | 0.55 |  | 0.04 | 0.68 |  | 0.73 | 0.36 |  | 343.8° | 64.6° |
| Daughters | -0.56 | 0.54 |  | 0.10 | 0.59 |  | -0.28 | 0.55 |  | 0.89 | 0.40 |  | 20.2° | 47.3° |
| Sons | 0.23 | 0.56 |  | 0.10 | 0.54 |  | -0.39 | 0.57 |  | 0.73 | 0.37 |  | 168.6° | 80.2° |

*Note.* P-T = People-Things axis; D-I = Data-Ideas axis; τ = profile elevation; α = profile differentiation; δ = profile orientation. M and SD of δ correspond to angular mean and angular variance as defined by Mardia & Jupp (2000).

Supplemental Table 2

*Correlations between Circumplex Dimensions*

| Dyad Type | People-Things | Data-Ideas |
| --- | --- | --- |
| Mother-Father | .29^*^ | .41^*^ |
| Mother-Son | .17^*^ | .19^*^ |
| Mother-Daughter | .16^*^ | .24^*^ |
| Father-Son | .22^*^ | .19^*^ |
| Father-Daughter | .20^*^ | .17^*^ |

*^*^ p* < .05.

*
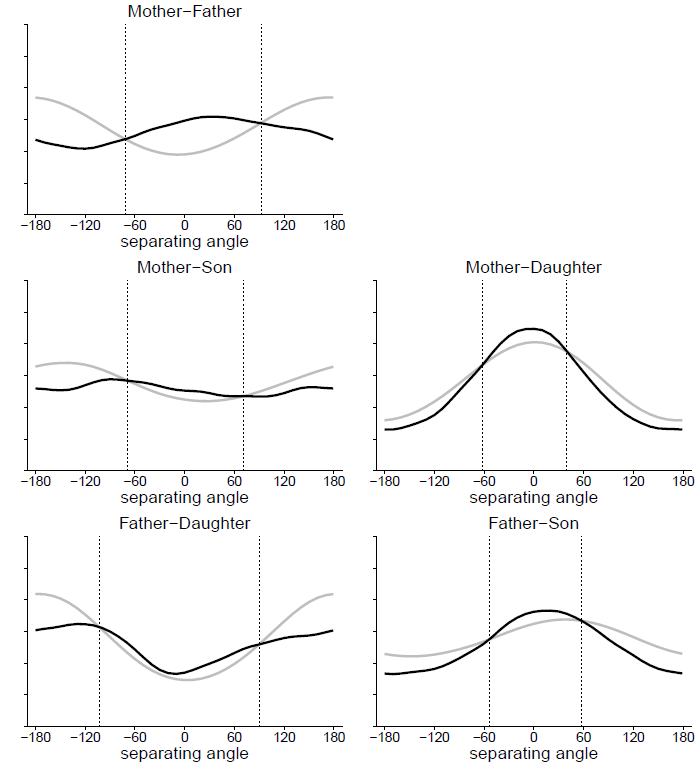
*

*Supplemental Figure 1.* Smoothed distributions of separating angles for real (black) and pseudo-families

(gray).
